# Levels of Human Immunodeficiency Virus DNA Are Determined Before ART Initiation and Linked to CD8 T-Cell Activation and Memory Expansion

**DOI:** 10.1093/infdis/jiz563

**Published:** 2019-11-28

**Authors:** Genevieve E Martin, Matthew Pace, Freya M Shearer, Eva Zilber, Jacob Hurst, Jodi Meyerowitz, John P Thornhill, Julianne Lwanga, Helen Brown, Nicola Robinson, Emily Hopkins, Natalia Olejniczak, Nneka Nwokolo, Julie Fox, Sarah Fidler, Christian B Willberg, John Frater

**Affiliations:** 1 Peter Medawar Building for Pathogen Research, Nuffield Department of Medicine, University of Oxford, Oxford, United Kingdom; 2 Big Data Institute, Li Ka Shing Centre for Health Information and Discovery, University of Oxford, Oxford, United Kingdom; 3 Division of Medicine, Wright Fleming Institute, Imperial College, London, United Kingdom; 4 Department of Genitourinary Medicine and Infectious Disease, Guy’s and St Thomas’ NHS Foundation Trust, London, United Kingdom; 5 Chelsea and Westminster Hospital, London, United Kingdom; 6 King’s College National Institute for Health Research Biomedical Research Centre, London, United Kingdom; 7 Imperial College National Institute for Health Research Biomedical Research Centre, London, United Kingdom; 8 National Institute of Health Research Biomedical Research Centre, Oxford, United Kingdom

**Keywords:** HIV, HIV reservoir, primary HIV infection, T cells

## Abstract

Initiation of antiretroviral therapy (ART) in early compared with chronic human immunodeficiency virus (HIV) infection is associated with a smaller HIV reservoir. This longitudinal analysis of 60 individuals who began ART during primary HIV infection (PHI) investigates which pre- and posttherapy factors best predict HIV DNA levels (a correlate of reservoir size) after treatment initiation during PHI. The best predictor of HIV DNA at 1 year was pre-ART HIV DNA, which was in turn significantly associated with CD8 memory T-cell differentiation (effector memory, naive, and T-bet^−^Eomes^−^ subsets), CD8 T-cell activation (CD38 expression) and T-cell immunoglobulin and mucin-domain containing-3 (Tim-3) expression on memory T cells. No associations were found for any immunological variables after 1 year of ART. Levels of HIV DNA are determined around the time of ART initiation in individuals treated during PHI. CD8 T-cell activation and memory expansion are linked to HIV DNA levels, suggesting the importance of the initial host-viral interplay in eventual reservoir size.

## Teaser

Human immunodeficiency virus (HIV) DNA persists within the viral reservoir despite therapy. A longitudinal analysis of 60 individuals treated during early HIV infection showed that HIV DNA levels are “set” early in infection and predicted by an activated immune response.

Human immunodeficiency virus (HIV) persists despite antiretroviral therapy (ART) in a reservoir of latently infected cells [1, 2], which is the focus of potentially curative interventions [3, 4]. There is much interest in which clinical, virological, or immunological parameters might determine the size of the reservoir.

There is evidence that T-cell immunity before ART initiation may be key to the formation of the HIV reservoir. Pretherapy CD4 and CD8 HIV-specific T-cell responses have been linked with lower levels of HIV DNA [5, 6] and T-cell activation has been shown in cross-sectional studies to relate to HIV reservoir size [5, 7–9]. In addition, immune checkpoint receptor (ICR) expression has been linked to reservoir size [5, 7, 8, 10, 11], however, a limitation of many of these studies is that ICR expression was measured on bulk T cells. Because the HIV reservoir is preferentially found in memory subsets [11, 12] that express higher levels of ICRs [10, 13–16], this may act as a potential confounder [17], and these studies often do not account for the expression of multiple ICRs and the relationship with T-cell activation [13–16, 18–20].

Accordingly, there are limited and conflicting data on which parameters predict reservoir size in treated HIV infection. Furthermore, few studies have assessed immunological factors during early HIV infection that may determine subsequent reservoir size [5, 6, 21]. In the current study, we aimed to clarify this by using a longitudinally studied cohort of individuals treated during primary HIV infection (PHI) who were sampled before ART and again 1 year later once viremia was suppressed.

## METHODS

### Participant Information

HIV Reservoir Targeting with Early Antiretroviral Therapy (HEATHER) is a prospective observational cohort study of individuals who begin ART within 3 months of HIV diagnosis during PHI (West Midlands—South Birmingham Research Ethics Committee reference no. 14/WM/1104). Individuals are considered to have PHI if they meet any of the following: HIV positive antibody test result within 6 months of a negative result, HIV antibody negative with positive polymerase chain reaction (PCR) (or positive p24 antigen result or viral load [VL]), recent incident test algorithm assay results consistent with recent infection, equivocal HIV antibody test result supported by a repeated test within 2 weeks showing a rising optical density, or clinical HIV seroconversion illness supported by antigen positivity. The time of seroconversion was estimated as the midpoint between the most recent negative or equivocal test result and the first positive result for those who met relevant criteria, the date of recent incident test algorithm assay minus 120 days for individuals in whom this assay result indicated primary infection, and the date of first positive test for all other participants. Individuals were excluded from this analysis if they had not achieved VL suppression to <50 copies/mL by 1 year.

### Flow Cytometry

Cryopreserved peripheral blood mononuclear cells were thawed and stained in Horizon Brilliant Stain Buffer (BD) containing all antibodies and Live/Dead Near-IR (Life Technologies) at 1:300 dilution and stained at 4°C for 30 minutes in Horizon. Panel 1 included the following: CD3 BV570 (UCHT1), CCR7 Pacific Blue (G043H7), and CD27 AlexaFluor700 (M-T271) (all BioLegend); CD4 BV605 (RPA-T4) and CD8 BV650 (RPA-T8) (BD); programmed cell death protein 1 (PD-1) phycoerythrin (PE)–eFluor610 (eBioJ105), CD45RA fluorescein isothiocyanate (HI100), and T cell immunoreceptor with immunoglobulin and ITIM domains (TIGIT) peridinin-chlorophyll protein complex (PerCP)–eFluor710 (MBSA43) (eBioscience); and Tim-3 PE (344823) (R&D).

Panel 2 included the following: CD3 BV570, CD38 AlexaFluor700 (HB-7) (BioLegend); CD4 BV605, CD8 BV650, PD-1 PE-eFluor610, and Tim-3 PE. After this, cells were washed twice before fixation and permeabilization with Foxp3 Buffer Set (BD). Staining for intracellular epitopes was performed with: T-bet fluorescein isothiocyanate (4B10) (BioLegend) and Eomes eFluor660 (WD1928) (eBioscience). Samples were acquired on an LSR II flow cytometer (BD). Data were analyzed using FlowJo software (version 10.8.0r1; Tree Star).

### HLA Typing

HLA typing was performed to intermediate resolution using PCR with sequence-specific primers.

### Soluble PD-1 and Tim-3 Quantification

Soluble PD-1 and soluble Tim-3 were measured in plasma by enzyme-linked immunosorbent assay (ELISA) using the Human PD-1 (PDCD1) ELISA kit (EHPDCD1; Thermo Fisher Scientific) and Quantikine ELISA Human TIM-3 Immunoassay kit (DTIM30; R&D Systems) at 1:2 and 1:5 dilutions, respectively.

### Total HIV DNA Quantification

CD4 T cells were isolated by negative selection using the EasySep Human CD4 Enrichment kit (Stemcell Technologies) to a purity of approximately 95%. DNA was extracted from CD4 T cells with the QiaAMP Blood Mini Kit (Qiagen). Cell copy numbers were quantified using albumin quantitative PCR; 25 000 cell equivalents were used in HIV DNA quantitative PCR with a probe targeted in the gag long terminal repeat (LTR) conserved region, performed in triplicate and as described elsewhere [22]. The mean number of copies of DNA was normalized to cell number and expressed as copies per 10^6^ CD4 T cells.

### Statistical Analyses

Analyses were performed using R (version 3.2.2 or 3.4.3) and GraphPad Prism (version 7.0b). Corrgrams were generated using the R package corrplot (version 0.84) with Spearman correlations. Boosted regression trees are a machine learning approach that builds a series of regression trees, with each subsequent tree iteratively aiming to improve the previous fit [23]. Boosted regression trees were fitted using the R package gbm3 (version 2.2). The algorithm hyperparameters were set to the following values: cross-validation folds, 10; interaction depth, 5; shrinkage/learning rate, 0.0005; bag fraction, 0.5; minimum terminal node observations, 5; and distribution, gaussian. The results were not sensitive to different values of the interaction depth parameter, and the shrinkage parameter was adjusted between 0.0001 and 0.001 to aim for the optimal number of trees (the number that minimized cross-validation error) to fall in the range of 3000–10 000. 

Results presented are summarized outcomes of 100 models. LASSO (least absolute shrinkage and selection operator) is a multivariable regression analysis method designed to cope with multicollinearity and large numbers of predictors by adding a penalty to the coefficient of each term. LASSO models [24] were fitted using the R package glmnet (version 2.0–16) [25]. Gaussian regression models were fitted with an additive linear model (no interactions); λ was the value that minimized 10-fold cross-validation error plus 1 standard error. Where data were imputed, this was performed using the R package MissForest; this was a single imputation with the model containing all parameters (as listed in Supplementary Table 1) [26]. 

## RESULTS

### Baseline Clinical Characteristics

We studied 60 individuals enrolled in HIV Reservoir Targeting with Early Antiretroviral Therapy (HEATHER), a longitudinal cohort of participants who began ART during PHI; clinical and demographic details are listed in Table 1. All participants were male and began ART a median (interquartile range) of 29 (14–47) days after HIV diagnosis and 49 (33–93) days after estimated seroconversion. Different methods for diagnosing PHI (Table 1) were used; 25 participants (42%) were p24 antigen positive without detectable antibodies, consistent with Fiebig stage I or II at the time of diagnosis.

**Table 1.** Demographic and Baseline Clinical Characteristics of Participants^a^

Characteristic	Participants, No. (%)^b^
Male sex	60 (100)
Age, median (IQR), y	34 (28–41)
Interval median (IQR), d	
From confirmed HIV-positive test to ART initiation	29 (14–47)
From estimated date of seroconversion to ART initiation	49 (32–93)
From ART initiation to first VL <50 copies/mL^c^	133 (90–230)
Baseline values, median (IQR)	
CD4 T-cell count, cells/μL^d^	530 (409–663)
CD8 T-cell count, cells/μL^d^	1037 (837–1318)
CD4/CD8 ratio^d^	0.5 (0.4–0.8)
VL, log_10_ copies HIV RNA/mL	5.4 (4.5–6.4)
Method for diagnosing primary HIV infection	
Antigen positive (p24 or PCR) but antibody negative	25 (42)
Rising antibody titer	1 (2)
Negative test result within 6 mo of positive result	28 (47)
Recent incidence testing algorithm	6 (10)
Mode of acquisition	
MSM	54 (90)
MSW	1 (2)
Unknown/unrecorded	5 (8)
Initial ART regimen	
Unknown/unrecorded	3 (5)
Backbone	
Tenofovir containing	52 (87)
Abacavir containing	5 (8)
Additional agent(s)	
Protease inhibitor	33 (55)
NNRTI	11 (18)
Integrase inhibitor	12 (20)
Protease inhibitor plus integrase inhibitor	1 (2)

Abbreviations: ART, antiretroviral therapy; HIV, human immunodeficiency virus; MSM, men who have sex with men; MSW, men who have sex with women, NNRTI, nonnucleoside reverse-transcriptase inhibitor; PCR, polymerase chain reaction; VL, viral load.

^a^Demographic and baseline clinical characteristics of included participants from the HEATHER cohort.

^b^Data represent no. (%) of participants unless otherwise identified as median (IQR) values.

^c^Fifty-nine of 60 individuals were virologically suppressed to <50 copies/mL before 1-year study visit; the remaining individual achieved virological suppression shortly thereafter, at the next VL measurement (Supplementary Figure 1)

^d^Data available for 59 of 60 individuals.

**Figure 1.**
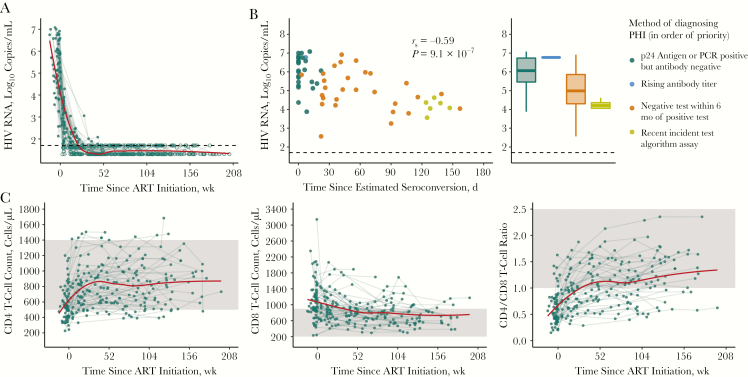
Measures of clinical progression during treated primary human immunodeficiency virus (HIV) infection. *A,* Viral load (VL) in the 4 years after antiretroviral therapy (ART) initiation (n = 60). Exact values are shown as closed circles, and those below the limit of detection as open circles; black dashed line indicates 50 copies/mL. *B,* Baseline VL relative to the number of days this was measured after estimated seroconversion (*left panel*; Spearman correlation) with the same data as box plots stratified by the method of diagnosing primary HIV infection (PHI) (*right panel*). If individuals met multiple diagnostic criteria, they are plotted as the criterion with the most reliability in estimating date of seroconversion; these are shown in the legend in the order of priority used. *C,* CD4 and CD8 T-cell counts and CD4/CD8 T-cell ratio in the 4 years after ART initiation (n = 63); the shaded region shows the normal range for these parameters. For *A* and *C,* a trend line (*red*) has been fitted using local polynomial regression fitting (LOESS) smoothing with an α value of .75. Abbreviation: PCR, polymerase chain reaction.

The participants had a high median baseline VL (5.4 log_10_ copies/mL; interquartile range, 4.5–6.4 copies/mL), which declined during ART (Figure 1A); the frequency of VL sampling and time to suppression are shown in Supplementary Figure 1. There was a relationship between the first measured (baseline) VL and the method used to diagnose PHI (Figure 1B). Baseline VL was higher when measured closer to estimated seroconversion (*r*_*s*_ = −0.59; *P* = 9.1 × 10^−7^), suggesting that VL is of limited utility as a predictive variable in PHI because a stable “set point” has not yet been reached. The dynamics of CD4 and CD8 T-cell counts, as well as CD4/CD8 T-cell ratio after ART initiation are shown in Figure 1C.

### Pre-ART HIV DNA is Predictive of HIV Reservoir Size After 1 Year of ART

**Figure 2.**
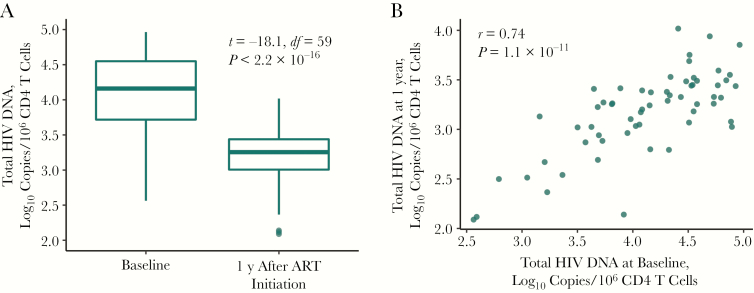
Total human immunodeficiency virus (HIV) DNA levels during treated primary HIV infection, showing relationship between total HIV DNA levels measured at baseline and 1 year after antiretroviral therapy (ART) initiation (n = 60). Comparisons were made using paired *t* tests (*A*) and Pearson correlation (*B*).

Quantitation of HIV DNA (copies per 10^6^ CD4 T cells) is used here as the surrogate measure of reservoir size. Compared with pre-ART levels, HIV DNA levels decreased a mean of 0.9 log_10_ copies after 1 year of therapy (Figure 2A) (*P* < 2.2 × 10^−16^). HIV DNA levels before therapy and after 1 year of ART were highly correlated (Figure 2B) (*r* = 0.74; *P* = 1.1 × 10^−11^). For a subset of 17 individuals, levels of total HIV DNA were also available 3 years after ART initiation, and had declined a further 0.3 log_10_ copies since year 1. (HIV DNA levels were not correlated between those 2 measurements, although a positive trend was evident [Supplementary Figure 2] [*r *= 0.41; *P* = .10]).

### Immunological and Clinical Variables Associated With HIV DNA Level

**Figure 3.**
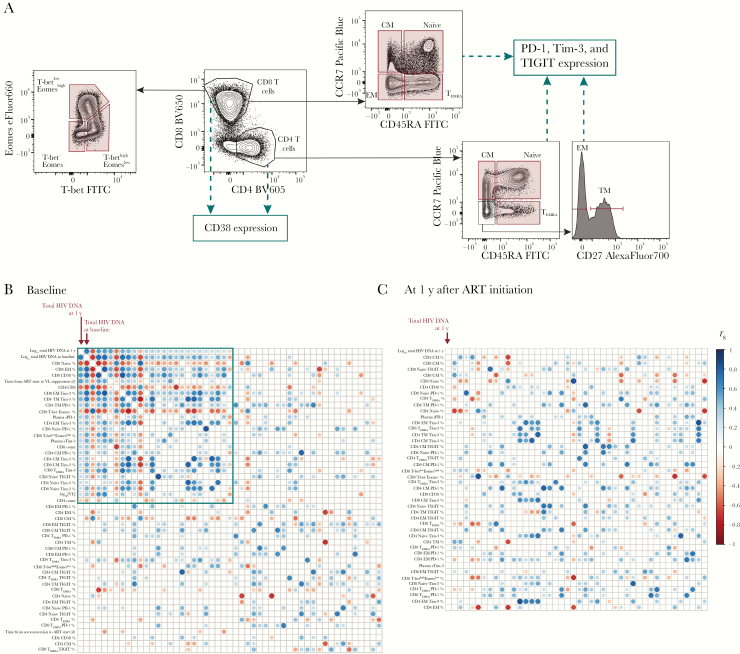
Immunological and clinical variables associated with human immunodeficiency virus (HIV) reservoir size are highly correlated with one another. *A,* Schematic showing the T-cell subsets and surface markers measured by flow cytometry in this analysis. The frequency of populations gated in red was included in analysis, as well as the expression of CD38, PD-1, Tim-3 and TIGIT on populations marked. Further gating details are shown in Supplementary Figure 3. *B, C,* Correlations between clinical or immunological variables and HIV reservoir size. Corrgrams show the relationship between HIV reservoir size at 1 year (log_10_ total HIV DNA) and immunological or clinical variables (n = 60) measured at baseline (*B*) or after 1 year of antiretroviral therapy (ART) (*C*). The same immunological variables were included at both time points, and clinical variables at baseline only. Reservoir size at 1 year (log_10_ total HIV DNA) is shown in the top left corner and is marked. For both *B* and *C,* variables have been ranked based on the magnitude of absolute correlation coefficient with log_10_ total HIV DNA at 1 year in decreasing order from the top left corner. The size and color of each circle correspond to the correlation coefficient between any 2 variables. Correlation coefficients were calculated using the Spearman method with pairwise complete observations; only correlations significant at the .05 level are shown (other boxes are left blank). The green box encloses variables that are significant correlated with 1 year log_10_ total HIV DNA at 1 year (at the .05 level). Abbreviations: CM, central memory; EM, effector memory; EMRA, effector memory T-cells re-expressing CD45RA; FITC, fluorescein isothiocyanate; PD-1, programmed cell death protein 1; sPD-1, soluble PD-1; sTim-3, soluble Tim-3; TIGIT, T cell immunoreceptor with immunoglobulin and ITIM domains; Tim-3, T cell immunoglobulin and mucin-domain containing protein 3; TM, transitional memory; VL, viral load.

We next explored which clinical and immunological variables predicted HIV reservoir size (as listed in Supplementary Table 1). Clinical variables measured were CD4 and CD8 T-cell counts, VL, CD4/CD8 T-cell ratio, time to ART start, and time to VL suppression with ART. Immunological measures included flow cytometric quantitation (Figure 3A and Supplementary Figure 3) of CD4 and CD8 T-cell memory subsets, CD38 expression, ICR expression (PD-1, TIGIT, and Tim-3 on memory CD4 and CD8 T cells), and soluble plasma ICRs (soluble PD-1 and soluble Tim-3). T-bet and Eomes are transcription factors that operate in concert in the development of effector T-cell functions, with a T-bet^dim^Eomes^high^ CD8 T-cell phenotype linked to functional exhaustion during HIV infection [27]; the proportion of T-bet/Eomes expressing CD8 populations was also measured.

Several parameters were highly correlated with HIV DNA levels. Corrgrams were used to screen the relationship of variables measured before ART initiation (baseline; Figure 3B) and after 1 year of ART (Figure 3C), with the HIV reservoir at 1 year. Each row or column in the corrgram represents a different variable ordered by the strength of the Spearman correlation with reservoir size at 1 year (in the top left corner). Circles indicates correlations between 2 variables (*P* < .05). Variables with a statistically significant relationship to reservoir size at 1 year are indicated in Figure 3B (*green box*).

The corrgrams for variables at baseline and 1 year look very different (Figure 3B and 3C, respectively). When exploring variables measured immediately before ART, those that were closely related to reservoir size (top left corner of corrgram) were also highly correlated with each other. The variable with the strongest correlation with HIV reservoir size at 1 year was the level of HIV DNA measured at baseline. However, 25 other variables were associated with the reservoir and/or each other (Figure 3B*, green box*).

For variables measured after 1 year of ART—the same time the HIV reservoir was measured (Figure 3C)—there is little evidence of any correlation with reservoir size. These data suggest that certain variables are the key determinants of the HIV DNA level just before ART initiation, which is, in turn, the main predictor of the HIV reservoir later during ART.

### CD8 T-Cell Activation and Memory Expansion as Key Determinants of HIV DNA Level

The data set poses several challenges for multivariable models, especially because 7.4% of observations are missing (Supplementary Figure 4), owing, for example, to unavailable samples. The large number of parameters measured relative to observations was also problematic, as was the strong correlations between many of these variables. To ensure the robustness of any conclusions, 2 models with different approaches to prediction from complex data, boosted regression trees, and LASSO regression, were fitted.

**Figure 4.**
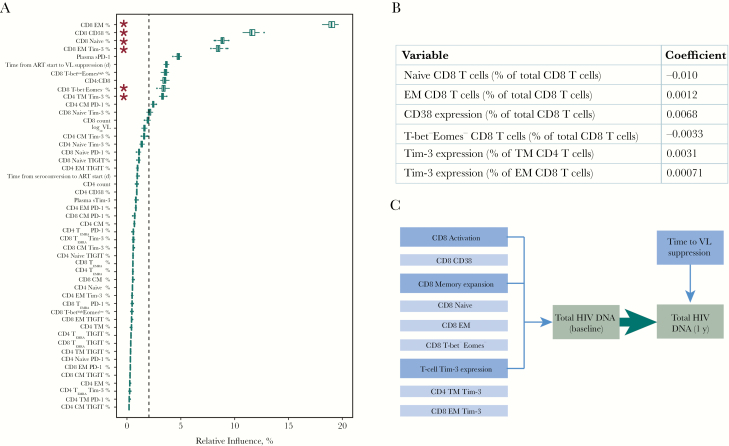
Immunological and clinical variables that relate to total human immunodeficiency virus (HIV) DNA. *A,* Boosted regression trees model to assess predictors of baseline total HIV DNA (49 predictors; n = 60); box plots show the summary of 100 model runs. Influential predictors were defined as those whose relative contribution was >100, divided by the total number of covariates; this value is indicated by dashed vertical line. Asterisks denote variables that are also selected in *B*. *B,* Least absolute shrinkage and selection operator (LASSO) output for predictors of baseline total HIV DNA (49 predictors; n = 60; deviance explained, 0.62). Variables that do not significantly contribute to the model have a coefficient of 0; only those with a nonzero coefficient are shown, and missing values were imputed. Coefficients represent the change in log_10_ total HIV DNA per 1% higher predictor variable. *C,* Schematic illustrating which factors were associated with HIV DNA level during treated primary HIV infection in this analysis. Abbreviations: ART, antiretroviral therapy; CM, central memory; EM, effector memory; EMRA, effector memory T cells re-expressing CD45RA; PD-1, programmed cell death protein 1; sPD-1, soluble PD-1; sTim-3, soluble Tim-3; TIGIT, T cell immunoreceptor with immunoglobulin and ITIM domains; Tim-3, T cell immunoglobulin and mucin-domain containing protein 3; TM, transitional memory; VL, viral load.

A boosted regression trees model was fitted with baseline total HIV DNA as the outcome, and all other baseline variables as predictors (Figure 4A). The figure shows that 11 of the 49 predictors had a consistent influence in predicting baseline reservoir size. Notably, CD8 memory subsets (the proportions of effector memory and naive cells) and CD8 CD38 expression were the variables with the highest relative influence.

LASSO regression identified 6 variables that were independently predictive of baseline HIV DNA (Figure 4B); all 6 were also selected by the boosted regression trees model. The variables with greatest influence on baseline HIV DNA levels were associated with CD8 memory expansion (the proportion of naive and effector memory as well as T-bet^−^Eomes^−^ CD8 T cells) and T-cell Tim-3 expression (effector memory CD8 and transitional memory CD4 T cells), as well as CD38 expression on CD8 T cells. A sensitivity analysis was conducted using only observations that were complete; results from both analyses were consistent, noting that the unimputed model selects fewer variables (Supplementary Table 2).

### Pre-ART HIV DNA Level as Dominant Predictor of Reservoir Size After 1 Year of ART

After establishing which variables were related to baseline HIV DNA, we fit regression models to explore whether any variables had additional relationships with reservoir size after 1 year of ART. HIV DNA at baseline was the most influential variable (Table 2, Supplementary Figure 5, and Supplementary Table 2), but no immunological variables measured at 1 year affected reservoir size, consistent with the modest correlations observed in the Figure 3C corrgram (model B in Table 2 and Supplementary Figure 5*B*). Of the clinical variables and immunological variables measured at baseline, only time from ART start to VL suppression independently predicted reservoir size (model A in Table 2 and Supplementary Figure 5*A*).

**Table 2.** Predictors of Reservoir Size at 1 Year^a^

Predictors	Coefficient	
	Model A	Model B
Baseline log_10_ total HIV DNA	0.29	0.27
Time from ART start to VL suppression (d)	0.00031	…

Abbreviations: ART, antiretroviral therapy; HIV, human immunodeficiency virus; VL, viral load.

^a^Least absolute shrinkage and selection operator (LASSO) output for predictors of reservoir size (total HIV DNA level at 1 year). Model A includes all baseline clinical and immunological variables, including baseline total HIV DNA levels (50 predictors; n = 60; deviance explained, 0.49). Model B includes all immunological measures at 1 year along with baseline total HIV DNA level (44 predictors; n = 60; deviance explained, 0.42). Variables that do not significantly contribute to the model have a coefficient of 0; only those with a nonzero coefficient are shown. Missing values were imputed. Coefficients represent the change in log_10_ total HIV DNA per unit predictor variable.

### Relationship Between Reservoir Size and HLA Class I

**Figure 5.**
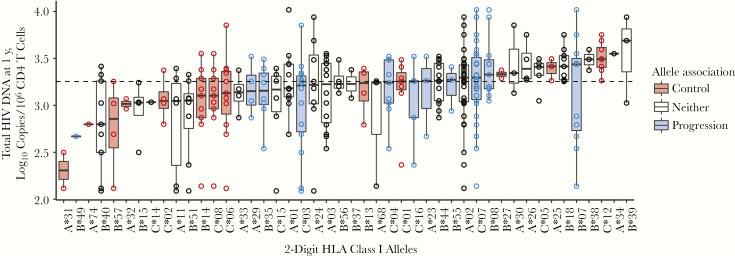
Relationship between HLA class I alleles and total human immunodeficiency virus (HIV) DNA 1 year after antiretroviral therapy (ART) initiation. Alleles, shown on the x-axis, are ordered based on the median value of total HIV DNA for all individuals possessing that allele. Box plots show the distribution of total HIV DNA among individuals possessing the allele, and each observation is shown as an open circle. Data are shown for 58 individuals and a total of 316 alleles. For 1 individual, only B alleles were available and are included here; for another, only A and C alleles were available. Where individuals were homozygous for a given allele, it is shown only once. Dashed line represents median value of total HIV DNA for the entire cohort. Alleles were classified as being associated with disease progression (*blue*), disease control (*red*), or neither (*white*), based on those identified in the International HIV Controllers Study at a significance level of .05 [28].

HLA type can be considered a surrogate marker of HIV-specific CD8 T-cell immunity [28, 29]. Figure 5 shows the relationship between HLA class I alleles and HIV DNA after 1 year of ART. Alleles associated with viral control cluster together, associated with low HIV DNA levels. The converse is seen for alleles associated with progression. The same relationship was observed with baseline HIV DNA (data not shown).

Our data are consistent with HIV specific immunity, the general immune landscape and clinical parameters all contributing to the size of the HIV reservoir with ART. Figure 4C summarizes our findings. The only 2 independent variables that predicted HIV reservoir size after 1 year of ART were pretherapy HIV DNA level and the time taken to achieve VL suppression after starting therapy. Baseline HIV DNA was associated with HLA class I type and specific markers of T-cell activation, expansion, and exhaustion.

## DISCUSSION

This work demonstrates the importance of immunological events before ART initiation in determining subsequent reservoir size. HIV DNA levels before ART were the most important predictor of HIV DNA a year later, suggesting that reservoir size is “set” early. HIV DNA before ART was more closely related to CD8 T-cell activation, memory expansion, and Tim-3 expression than any clinical parameters, including before ART (summarized in Figure 4C). Several predictors of reservoir size presented here have been observed previously [5, 30–33], but ours is the first study to define the independence of relationships between T-cell activation, memory expansion, and ICR expression with eventual reservoir size.

A key finding is that HIV DNA levels before ART initiation were the main predictor of subsequent reservoir size. Several other studies have shown a relationship between pretherapy HIV DNA levels and those during ART [30–33]; we extend these findings by demonstrating the dominance of pretherapy HIV DNA over contemporaneously measured T-cell parameters.

Higher levels of pre-ART T-cell activation predicted increased levels of HIV DNA. This might be driven by higher initial viral burden or poorer CD8 effector function. Cross-sectional studies have shown relationships between HIV DNA and CD8 T-cell activation when ART is already established [7, 9, 34], although this is not consistently seen [32, 35], and we did not find it in our study. Most prior studies have used HLA-DR/CD38 coexpression, which could explain this discrepancy. Indeed, the use of CD38 expression alone as a measure of CD8 T-cell activation is a limitation of the current study, because this marker is constitutively expressed on naive CD8 T cells [36].

Tim-3 is a marker of CD8 T-cell exhaustion during HIV infection [18]. Associated defects in proliferation, cytotoxicity, and the ability to rescue functionality via Tim-3 blockade, however, have been assessed only during the chronic phases of infection [18, 37]. In a 2018 analysis of exhaustion-specific genes during HIV infection, Tim-3 was not included because, like CD38, it was also highly expressed in effector as well as exhausted CD8 T-cell transcriptomic signatures [38]. That Tim-3 has activating roles is supported by findings that signaling through Tim-3 enhances TCR signaling in lymphocytic choriomeningitis virus infection models [39]. It is thus possible that during PHI Tim-3 marks a population of activated but not yet terminally exhausted cells, and that its link with HIV DNA content in this study represents T-cell activation.

We have focused on the relationship of T-cell expansion and ICR expression to the HIV reservoir. Immune activation during HIV infection is likely multifactorial—being due not only to the virus but to other processes (including microbial translocation and herpesvirus, particularly cytomegalovirus, coinfection) [40]. Innate immune responses have been linked to reservoir size and play a role in mediating this activation. 

A 2018 study of soluble biomarkers during acute infection demonstrated that several of these were related to HIV reservoir size (also measured by total HIV DNA) before and after 96 weeks of ART, independently of VL [21]. The soluble biomarkers identified can all be produced by myeloid cells in response to interferon α/γ signaling; the authors of that study speculate that this could indirectly reflect innate and/or T-cell responses to viral replication [21]. In addition, recent evidence suggests that immunometabolic pathways may have a relationship with the HIV reservoir [41, 42]. These factors, not measured in the current study, may play a role in mediating the observed relationship between T-cell expansion/activation and reservoir size, and they require further study.

It is interesting that VL was only modestly correlated with baseline HIV DNA level, because we had hypothesized that these would be closely linked. Several studies have demonstrated a relationship between pretherapy VL and HIV DNA [30, 32, 43, 44]. Notably, many of these studies included individuals treated during chronic infection, when VL will have reached set point. In contrast, during PHI the VL is substantially more labile. Within this cohort there was a link between baseline VL and how long after estimated seroconversion this was measured (Figure 1B), whereby individuals with more recent seroconversion had a higher VL. This has also been observed in another PHI cohort with similar time since infection [45], and the finding suggests that VL measures taken over this time capture the decline from peak, rather than a steady state, a finding that could explain the modesty of the relationship seen in our study.

It is also possible that the overall pre-ART viral burden (duration and magnitude of viremia) influences HIV reservoir size and is poorly captured by a single measurement during PHI. The consistent observation that earlier ART limits reservoir size [6, 31] implies a role for the total pretherapy viral burden. Two findings here suggest an influence of viral burden on overall reservoir size. The first is the previously reported relationship between HLA class I alleles and HIV reservoir size [5], which we confirmed, and which invokes a role for CD8 T-cell killing of virally infected cells as a driver of reservoir size. 

The second finding is the observation that time to VL suppression had an influence on reservoir size independently of baseline HIV DNA. This may be because individuals with longer time to VL suppression may have a window after ART initiation for reservoir seeding to continue, a hypothesis supported by recent findings from a large cohort study showing that time to suppression and viral blips influence HIV DNA levels [46]. Slower time to viral suppression may also reflect higher pretherapy viral burden. Although most individuals began ART at their baseline visit (median, 0 days after baseline visit; 82% within 1 week), a small proportion had a larger interval between these dates (maximum, 48 days), providing additional time for reservoir seeding not captured in the baseline HIV DNA measurement.

Several cross-sectional studies have shown a relationship between PD-1 or TIGIT expression on bulk CD4 T cells during ART and overall reservoir size [7, 8, 10, 47]. In contrast, we did not find this relationship. Previous studies, which have measured PD-1 on bulk T cells [7, 8, 10], may actually be capturing T-cell maturation, because the expanded memory subsets express higher levels of ICRs [10, 13–16], potentially explaining this discrepancy. Knowing that ICR expression on bulk T cells may be confounded by memory composition, we chose to measure these parameters on different memory subsets. Overall, this work suggests that memory expansion may be more closely linked to HIV DNA levels than ICR expression.

A limitation of the current study is that reservoir size was measured only using total HIV DNA. This measure is clinically relevant because lower levels have been associated with delayed viral rebound after treatment interruption [3]. Most of the HIV reservoir, however, is not replication competent [48, 49], and we did not assess whether these immunological measures had any impact on the quality of proviruses comprising the reservoir. A major strength of the study is its longitudinal design. Although 1 year of follow-up represents a significant duration, it may not be sufficient for ART to stabilize the HIV reservoir. Supporting the use of this time point, other studies have shown relationships between HIV DNA levels at early time points and those much longer after ART initiation [30, 32, 50]. However, understanding predictors of the eventual long-term reservoir will require further analyses after longer follow-up.

This work has shown that the magnitude of the early immunological insult, reflected in CD8 T-cell activation and memory expansion, drives HIV DNA levels. These results suggest that targeting of host or viral factors that lead to early viral expansion and T-cell activation may be a way of limiting HIV reservoir size, and they confirm the importance of starting ART as early as possible.

## Supplementary Data

Supplementary materials are available at *The Journal of Infectious Diseases* online. Consisting of data provided by the authors to benefit the reader, the posted materials are not copyedited and are the sole responsibility of the authors, so questions or comments should be addressed to the corresponding author.

jiz563_suppl_Supplementary_Table

## Notes

Presented in part: AIDS 2018: 22nd International AIDS Conference, Amsterdam, Netherlands, 23-27 July 2018. Abstract WEPEA027.

***Acknowledgments.*** We thank the participants in the HIV Reservoir Targeting with Early Antiretroviral Therapy (HEATHER) study. This study is conducted as part of the CHERUB (Collaborative HIV Eradication of Reservoirs: UK BRC) collaboration. The CHERUB Steering Committee includes Andrew Lever (University of Cambridge), Mark Wills (University of Cambridge), Jonathan Weber (Imperial College, London), Sarah Fidler (Imperial College, London), John Frater (University of Oxford), Lucy Dorrell (University of Oxford), Mike Malim (King’s College, London), Julie Fox (King’s College London), Ravi Gupta (University College London), and Clare Jolly (University College London). Thank you to the following who have been involved with the recruitment of HEATHER participants at the trial sites: Kristin Kuldanek, Heather Lewis, and Rebecca Hall (St Mary’s Hospital), Teresa Solano (St Thomas’ Hospital), and Sathya Visvendra, Rhian Bull, and Gabriele Pasluostaite (Chelsea & Westminster Hospital).

The raw data supporting the conclusions in this article will be made available by the authors on appropriate application. Data are from the HEATHER study, from which unrestricted release of data for public deposition would breach compliance with the protocol approved by the research ethics boards. Data can be accessed from the CHERUB steering committee by contacting the correspondence author (john.frater@ndm.ox.ac.uk).

***Author contributions.*** G. E. M. and J. Frater devised the study, analyzed data, and wrote the manuscript; F. M. S. and J. H. advised on the analysis of data; G. E. M., M. P., E. Z., J. P. T., H. B., N. R., E. H., and N. O. performed laboratory experiments; J. M. managed the patient cohorts and collated demographic data; J. P. T., J. L., N. N., J. Fox, and S. F. managed clinical sites and recruitment; and N. N., J. Fox, S. F., C. B. W., and J. Frater led on study design and management

***Disclaimer.*** The funders had no role in study design, data collection and analysis, decision to publish, or preparation of the manuscript.

***Financial support.*** This work was supported by the Medical Research Council (grant MR/L006588/1 to J. Frater) and the National Institute of Health Research Oxford Biomedical Research Centre.

***Potential conflicts of interest.*** All authors: No reported conflicts. All authors have submitted the ICMJE Form for Disclosure of Potential Conflicts of Interest. Conflicts that the editors consider relevant to the content of the manuscript have been disclosed..
