# Supplementary material for: Levels of Human Immunodeficiency Virus DNA Are Determined Before ART Initiation and Linked to CD8 T-Cell Activation and Memory Expansion
Source: J Infect Dis. 2019 Nov 28;221(7):1135–45. doi: 10.1093/infdis/jiz563 (PMC7075410; doi:10.1093/infdis/jiz563)
Supplement: jiz563_suppl_Supplementary_Table [file jiz563_suppl_supplementary_table.docx]

**Levels of HIV DNA are determined prior to ART initiation and linked to CD8 T cell activation and memory expansion**

**Supplementary material**

Genevieve E Martin, Matthew Pace, Freya M Shearer, Eva Zilber, Jacob Hurst, Jodi Meyerowitz, John P Thornhill, Julianne Lwanga, Helen Brown, Nicola Robinson, Emily Hopkins, Natalia Olejniczak, Nneka Nwokolo, Julie Fox, Sarah Fidler, Christian B Willberg, and John Frater on behalf of the CHERUB investigators

**List of contents:**Supplementary Figure 1. Viral load sampling frequency

Supplementary Figure 2. Reservoir size at 3 years post-ART initiation

Supplementary Figure 3. Representative gating of immunological subsets

Supplementary Figure 4. Missing immunological and clinical data

Supplementary Figure 5. Boosted regression tree results to assess the relative influence of predictors of reservoir size at 1 year

Supplementary Table 1. Parameters measured as part of this study

Supplementary Table 2. LASSO models using data without imputation

**Supplementary Figure 1.** Viral load sampling frequency


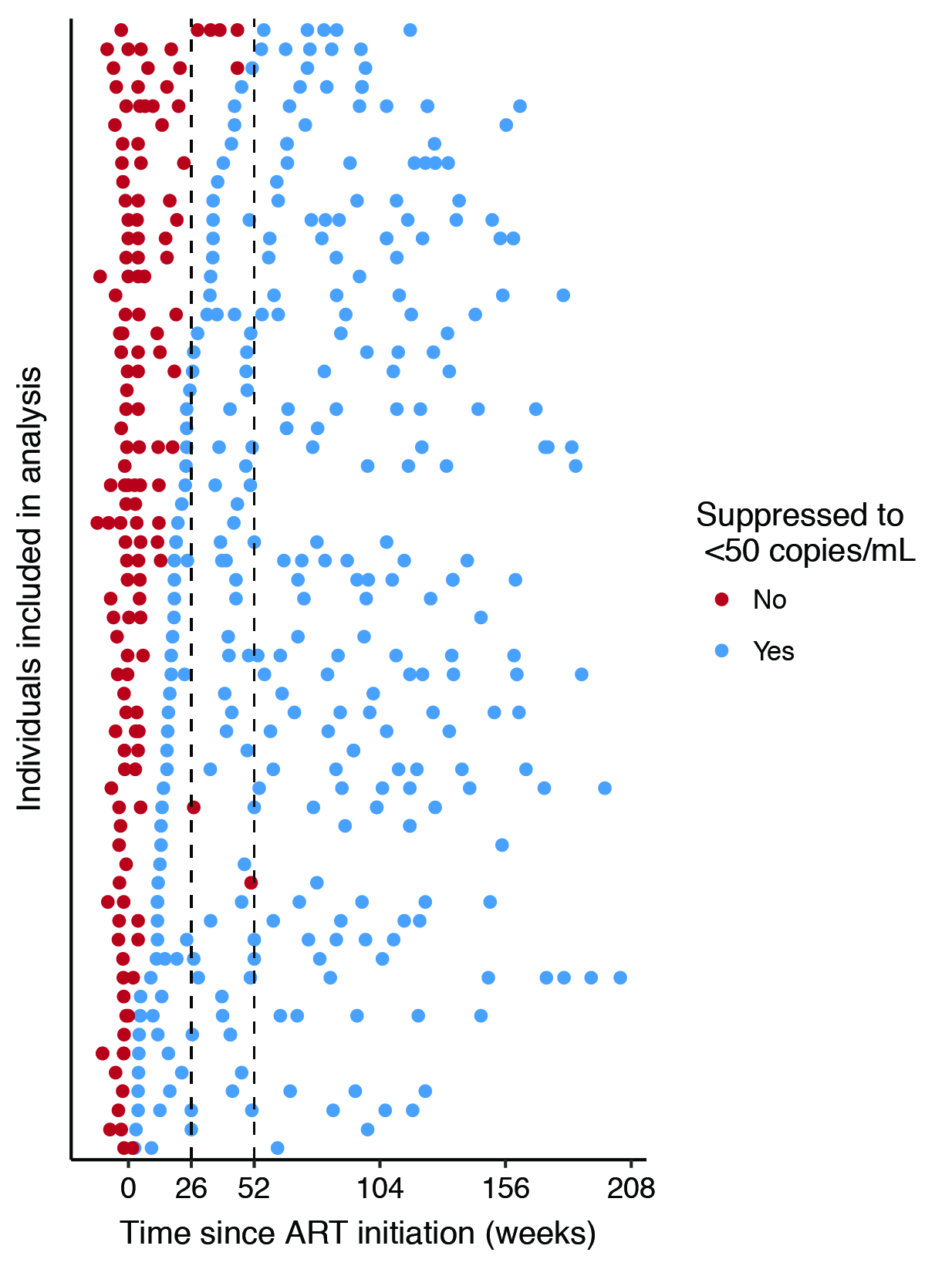


The viral load sampling frequency in individuals (n=60) included in the analysis. Each VL measurement is plotted at the time following ART initiation that it was taken and coloured according to whether the VL value was suppressed to <50 copies/mL (red if not suppressed, blue if suppressed). Individuals are ordered based on the time to viral load suppression (first measurement <50 copies/mL).

**Supplementary Figure 2.** Reservoir size at 3 years post-ART initiation


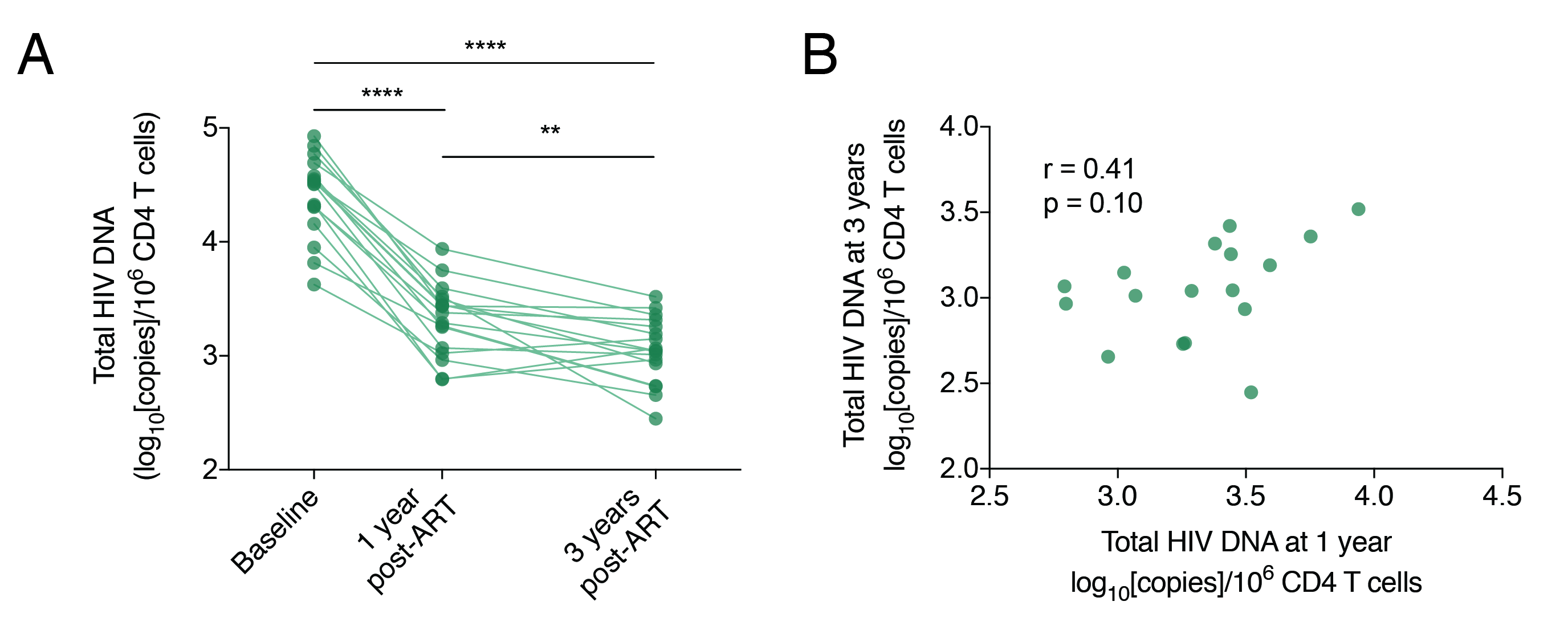


Relationship between total HIV DNA measured at baseline, 1 year following ART initiation and 3 years following ART initiation for a subset of individuals (n=17). For **(A)** comparison was made using an ANOVA (with pairing; overall p<0.0001) with post-hoc testing using Holm-Sidak’s multiple comparison test. ** indicates p <0.01, **** indicates p<0.0001. **(B)** a Pearson's correlation was performed.

**Supplementary Figure 3.** Representative gating of immunological subsets


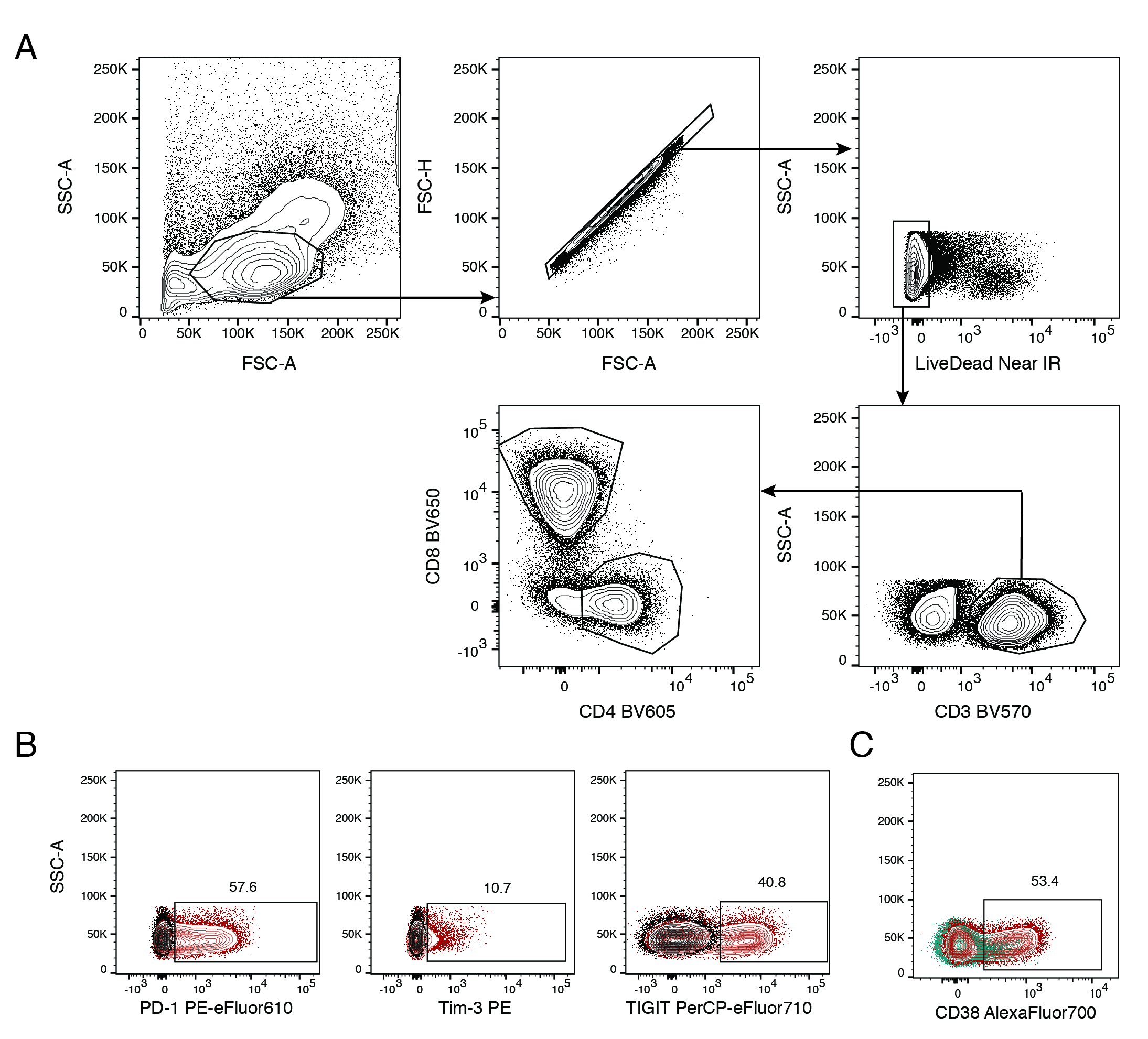


(A) Representative gating of CD4 and CD8 T cells. Cells were gated based on FSC-A vs SSC-A characteristics. Following doublet and dead cell exclusion, CD3 T cells were gated against SSC-A. CD4 and CD8 T cells were then gated based on the reciprocal expression of these markers. The subdivision of these populations further is shown in Figure 3A. (B) Representative gating of PD-1, Tim-3 and TIGIT plots are gated on CD8 EM cells and show staining from one individual during PHI (red) overlaid over gating control (black). (C) Representative gating of CD38 on CD8 T cells from one individual during PHI (red) overlaid over a healthy control (green).

**Supplementary Figure 4.** Missing immunological and clinical data


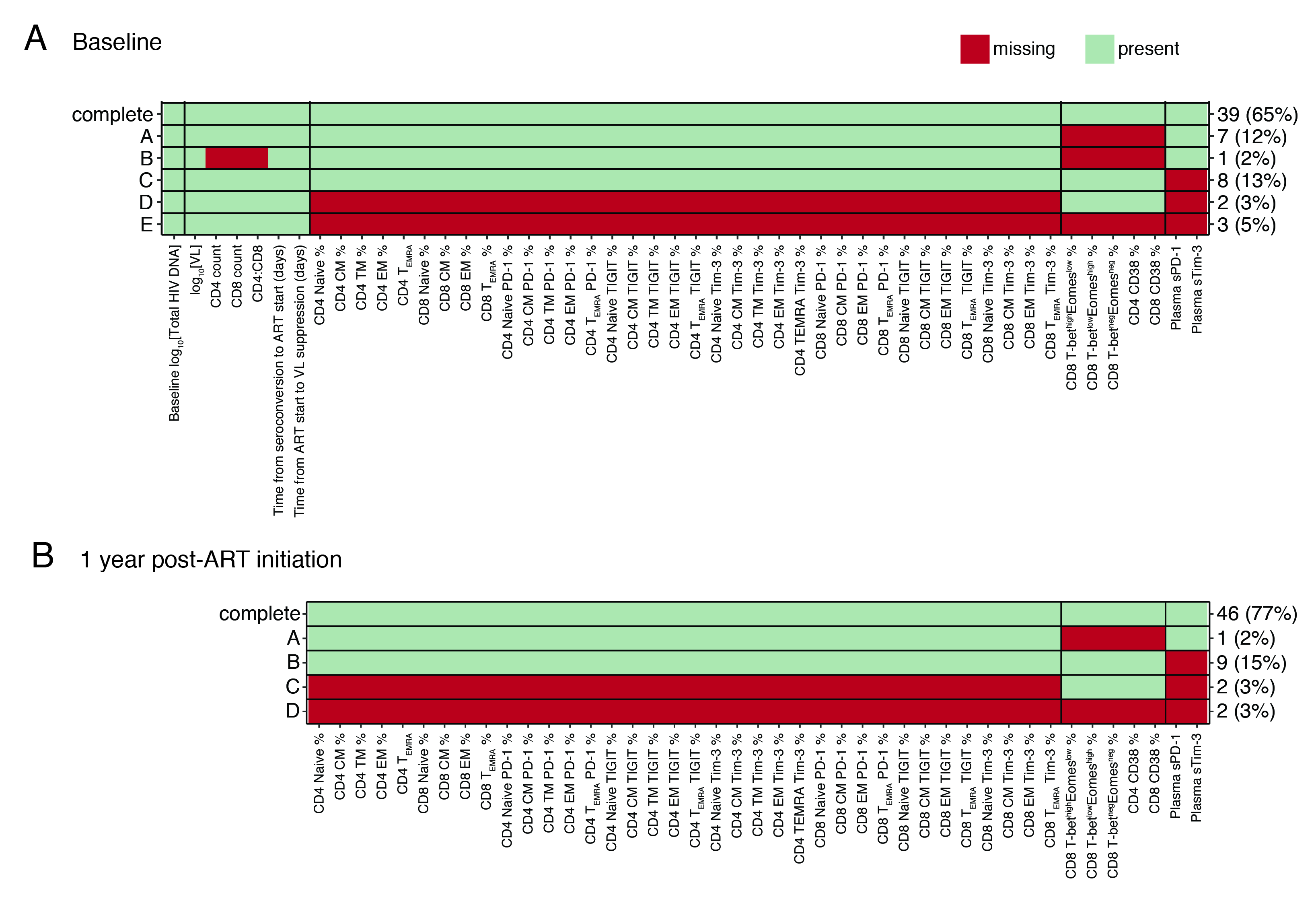


Missing immunological and clinical data. This diagram shows the patterns of missing data at baseline **(A)** and following 1 year of ART **(B)** for the 60 individuals included in analyses presented. Each row represents a pattern of data missingness and the right-hand side shows how many individuals have this pattern. For some individuals there are no immunological observations available at a given time point. These are Pattern E (n=3) at baseline (all three samples had very low viability upon thawing and were not able to be included) and Pattern D (n=2) at 1 year (these individuals had not returned for 1 year visits at the time the assays were performed).

**Supplementary Figure 5.** Boosted regression trees model results to assess the relative influence of predictors of reservoir size at 1 year


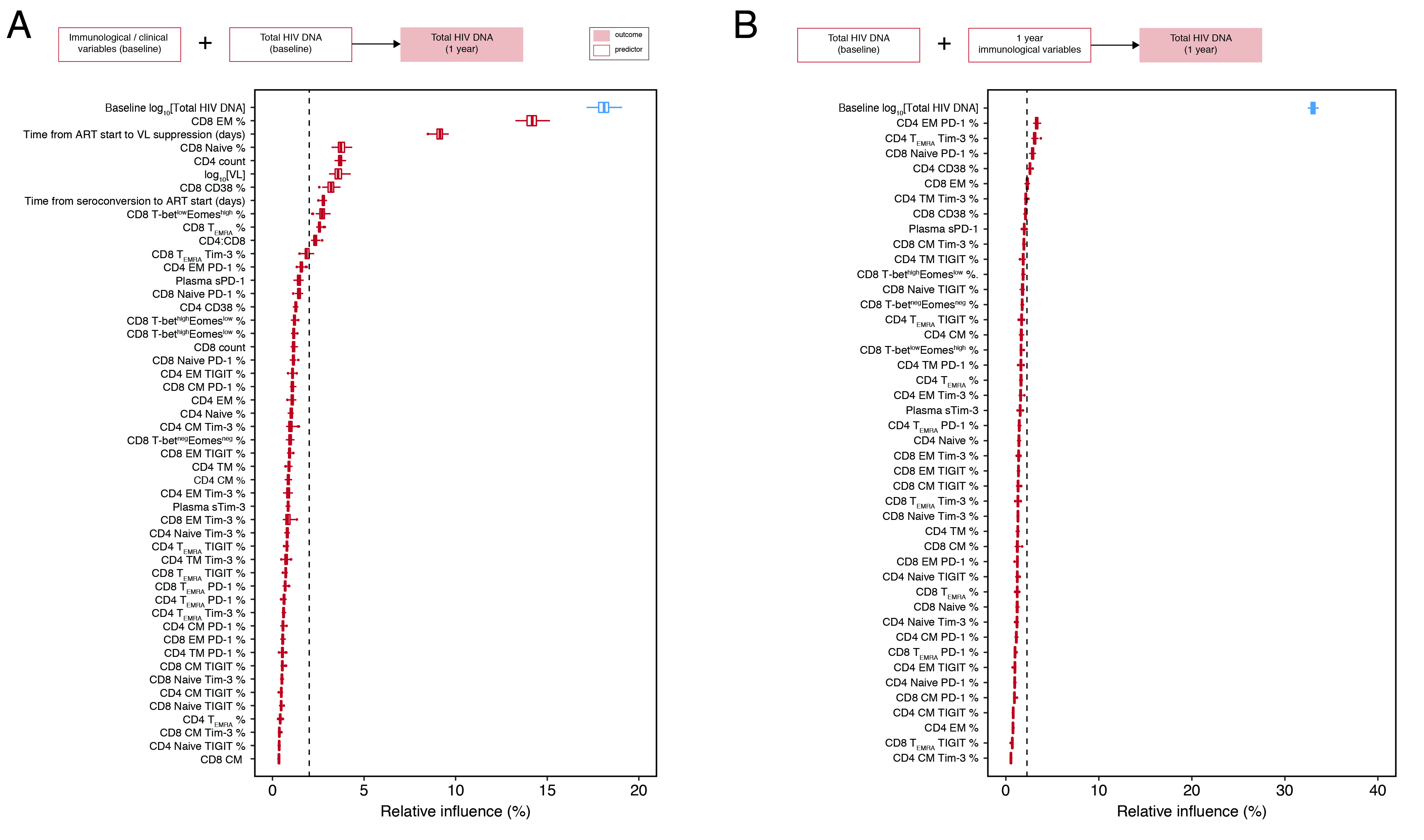


Boosted regression trees models to assess the relative influence of predictors of reservoir size (1 year total HIV DNA). **(A)** Includes all baseline clinical and immunological variables (50 predictors, n=60). **(B)** Includes all immunological measures at 1 year (1 year; 44 predictors, n=60). In both models, total HIV DNA at baseline was included as a predictor and is highlighted in blue. Boxplots show the summary of 100 model runs. Influential predictors were defined as those whose relative contribution was greater than 100 divided by the total number of covariates, this value is indicated by the dashed vertical line.

**Supplementary Table 1.** Parameters measured as part of this study

| Parameters | Technique | Measured at baseline | Measured at 1 year |
| --- | --- | --- | --- |
| Total HIV DNA | qPCR | Yes | Yes |
| Log_10_[VL]  CD4 count  CD8 count  CD4:CD4 ratio  Time from seroconversion to ART start (days)  Time from ART start to VL suppression (days) | Clinical data | Yes | No |
| CD4 Naïve %  CD4 CM %  CD4 TM %  CD4 EM %  CD4 T_EMRA_ %  CD8 Naïve %  CD8 CM %  CD8 EM %  CD8 T_EMRA_ %  CD4 Naïve PD-1 %  CD4 CM PD-1 %  CD4 TM PD-1 %  CD4 EM PD-1 %  CD4 T_EMRA_ PD-1 %  CD4 Naïve Tim-3 %  CD4 CM Tim-3 %  CD4 TM Tim-3 %  CD4 EM Tim-3 %  CD4 T_EMRA_ Tim-3 %  CD4 Naïve TIGIT %  CD4 CM TIGIT %  CD4 TM TIGIT %  CD4 EM TIGIT %  CD4 T_EMRA_ TIGIT %  CD8 Naïve PD-1 %  CD8 CM PD-1 %  CD8 EM PD-1 %  CD8 T_EMRA_ PD-1 %  CD8 Naïve Tim-3 %  CD8 CM Tim-3 %  CD8 EM Tim-3 %  CD8 T_EMRA_ Tim-3%  CD8 Naïve TIGIT %  CD8 CM TIGIT %  CD8 EM TIGIT %  CD8 T_EMRA_ TIGIT %  CD8 T-bet^high^Eomes^low^ %  CD8 T-bet^low^Eomes^high^ %  CD8 T-bet^neg^Eomes^neg^ %  CD4 CD38 %  CD8 CD38 % | Flow cytometry | Yes | Yes |
| Plasma sPD-1  Plasma sTim-3 | ELISA | Yes | Yes |

Complete list of parameters measured as part of this study.

**Supplementary Table 2.** LASSO models using data without imputation

| Corresponding model | Fig. 4C | Table 2 – Model A | Table 2 – Model B |
| --- | --- | --- | --- |
| Outcome | Baseline total HIV DNA | 1 year total HIV DNA | 1 year total HIV DNA |
| Predictors included | Baseline clinical and immunological variables | Baseline clinical and immunological variables | 1 year immunological variables |
| n | 39 | 39 | 46 |
| Deviance explained | 0.64 | 0.51 | 0.42 |
|  |  | *Coefficient* | *Coefficient* |
| Naive CD8 T cells (% of total CD8 T cells) | -0.016 | - | - |
| CD38 expression (% of total CD8 T cells) | 0.0062 | - | - |
| Tim-3 expression (% of TM CD4 T cells) | 0.0024 | - | - |
| Baseline log_10_[total HIV DNA] | N/A | 0.27 | 0.25 |
| Time from ART start to VL suppression (days) | - | 0.00046 | N/A |

Least absolute shrinkage and selection operator (LASSO) output for predictors of total HIV DNA. LASSO models presented here were constructed excluding any observations with missing variables (complete cases only) rather than imputing these missing values. Coefficients represent the change in log_10_[Total HIV DNA] per unit predictor variable. Variables which do not significantly contribute to the model have a coefficient of zero; only those with a non-zero coefficient are shown. N/A means that variable was not included in model construction.
